# Informal caregivers’ profile needs: where do we stand?

**DOI:** 10.1093/eurpub/ckac131.034

**Published:** 2022-10-25

**Authors:** A Henriques, D Sousa Loura, P Nogueira, A Costa

**Affiliations:** 1 Centro de Investigação, Inovação e Desenvolvimento em Enfermagem de Lisboa, Escola Superior de Enfermagem de Lisboa, Lisbon, Portugal; 2 Isntituto de Saúde Ambiental, Faculdade de Medicina da Universidade de Lisboa, Lisbon, Portugal; 3 Hospital Dona Estefânia, Centro Hospitalar Universitário de Lisboa Central, E.P.E., Lisbon, Portugal; 4 Escola Superior de Enfermagem de Lisboa, Lisbon, Portugal; 5 Laboratório de Biomatemática, Instituto de Medicina Preventiva e Saúde, Faculdade de Medicina da UL, Lisbon, Portugal

## Abstract

**Background:**

Non-communicable diseases’ increase and demographic ageing require a solution to manage long-term care (1), where informal caregivers are key actors (2). Optimization in policies designed to support their caregiving role is needed. Portugal is an aged country with high prevalence of family that take care of dependent relatives. ‘Informal caregivers’ profile in Lisbon County: a health community approach’ is a nurse-led research project designed to meet these challenges with the main aim: to develop a profile on informal caregivers in Lisbon county.

**Methods:**

Reporting the descriptive phase, a survey containing health/social questions was submitted to a non-probabilistic representative sample of careers, aged 18 years old or above in about 300 caregiver’s caring dependent persons resident in Lisbon, in 2021. Univariate descriptive analysis was performed.

**Results:**

Married and retired women’ caring for a parent were the most typical informal caregiver profile (n = 13, 4%). The majority do not have support from social services (n = 209, 61%). Two thirds live with the cared-for person (n = 219, 64%). Almost half (n = 150, 44%) have a university degree and only few planned the transition to a caregiver role (n = 13, 4%). No more than 10% had access to support programs (n = 71, 20%).

**Conclusions:**

Caregivers’ unmet needs can complexify societal mechanisms relying on their work. Addressing these needs will be key to develop a strategy focused on supporting informal caregivers’ priorities.

References

1. Zigante, V. (2018). Informal care in Europe: exploring formalisation, availability and quality. Publications Office of the European Union. https://doi.org/10.2767/78836.

2. Crisp, N. (Coord.) (2014). Um futuro para a saúde - todos temos um papel a desempenhar. Fundação Calouste Gulbenkian. https://www.gulbenkian.pt

**Key messages:**

• Caregivers profile needs will be key support a strategy on informal caregivers’ priorities.

• No more than 10% of informal caregivers in a relevant European County had access to support programs.

